# The usage of family audiobooks as a legacy for grieving children — an exploratory quantitative analysis among terminally ill parents and close persons

**DOI:** 10.1007/s00520-024-08945-x

**Published:** 2024-10-25

**Authors:** Gülay Ateş, Michaela Hesse, Henning Cuhls

**Affiliations:** 1 Institute for Digitalization and General Medicine, Center for Rare Diseases Aachen (ZSEA), Medical Faculty, RWTH Aachen University, Pauwelsstr. 30, 52074 Aachen, Germany; 2 Department of Palliative Medicine, University Hospital Bonn, Venusberg Campus 1, 53127 Bonn, Germany

**Keywords:** Terminally ill parent, Dependent children under the age of 18, Biographical work, Family audiobook, Digital legacy

## Abstract

**Background:**

Since 2017, terminally ill parents with dependent children under the age of 18 have been able to record an audiobook for their dependent children. This service allows them to narrate how they would like to be remembered in their voice. The family audiobook is a professionally supported, voluntary, free service that is unique in Germany. There is little research on digital memories for children. The study aims to understand how this service is used and its influence on children through responses of terminally ill parents and close persons.

**Methods:**

An anonymous online survey, accessible between September 2023 and November 2023, was conducted among terminally ill parents and their close persons with support from the Family Audiobook Association in Germany. Analyses were carried out using SPSS.

**Results:**

186 respondents, 95 terminally ill parents, and 91 close persons completed the online survey. Almost all terminally ill parents felt eased to have recorded a family audiobook. The two groups showed differences in how they used the family audiobook and how often they listened to it. While some children listen to the family audiobook with their bereaved parents or friends, other children are not yet ready for this, according to the open-ended responses of terminally ill parents and close persons.

**Conclusions:**

The family audiobook provides a valuable opportunity for terminally ill parents with dependent children under the age of 18 to tell their own biographical story, offer support to the bereaved in remembering, and preserve the voice of the deceased for the children. In addition, this approach could help healthcare professionals to reduce the stress associated with providing end-of-life care for terminally ill parents.

**Supplementary Information:**

The online version contains supplementary material available at 10.1007/s00520-024-08945-x.

## Introduction

In Germany, 50% of deaths in the 25–50 age group were caused by cancer in 2021 [1]. The terminally ill patients in this age group are at different stages of their lives. One example is the care of terminally ill parents with dependent children under the age of 18, which can present a significant challenge for patients, dependent children, and healthcare professionals due to the near-death nature of the disease trajectory in the patient’s life [2–8]. The advanced care planning process, which is designed to be patient-centered and tailored to the specific needs and life planning of patients with incurable cancer, plays a crucial role in the management of these patients [9–11]. Accordingly, in addition to medical, nursing, or treatment pathways, there are further priorities for a significant number of terminally ill parents with dependent children under the age of 18 [1, 12, 13].

Biographical interventions have been demonstrated to positively impact patients in palliative care, including improvements in quality of life, reductions in depression, and enhanced coping mechanisms to navigate life limitations. These limitations encompass not only the challenges of a life-limiting illness but also the physical and mental constraints that accompany it [14–16]. In the field of gerontology and geriatrics, interventions such as Dignity Therapy, Life Review, and Reminiscing have been demonstrated to have a positive impact on the quality of life in patients facing end-of-life or coping with life-threatening diseases [16]. A meta-analysis of scientific studies has shown that there are no standardized definitions and/or procedures for these types of conversations as an intervention [16]. However, Dignity Therapy addresses individual experiences, values, and hope in the context of life events to regain self-control and self-advocacy. Life Review focuses on self-evaluation and positive feelings about achievements and challenges through structured conversations about good and bad life events. Reminiscence also aims to prepare for one’s death as a conversation about positive and negative life events or meaning in life in terms of sharing memories, lullabies, and identity-forming areas of life. Structured conversations about life events and areas of meaning in life, as well as an evaluation of one’s achievements and failings, facilitate the formation of interpersonal connections and the development of shared experiences. The potential for a deeper intergenerational understanding of parental behavior is facilitated by the opportunity to reflect on past experiences and problem-solving strategies [14].

Keeping memories alive is a key issue of reminiscence interventions. In families with a terminally ill parent, children have little or no time to collect memories with their parents, so they rely on stories from others. The statements of bereaved parents and children also highlight the importance of preserving memories associated with a deceased parent [17–21]. The findings indicate that bereaved parents expressed their difficulties in taking responsibility for the memory of the deceased parent and in providing the children with letters from the deceased as a reminder [17, 18]. Those who experience bereavement often feel a sense of burden, especially as the bereaved parent takes on roles and responsibilities typically associated with the deceased parent. On the one hand, questions about the deceased parent during the grieving parent’s daily activities create a sense of pressure and overwhelm, and on the other hand, it is they, the grieving parents, who have an interest in keeping the memories alive [17, 18]. A legacy is an opportunity to learn first-hand about a deceased parent through letters, pictures, videos, or a family audiobook. Therefore, transferring some of the memories into digital formats could help to find an appropriate way of keeping memories alive for children. Bereaved children may benefit from having a video or audio recording of the deceased parent, which “could capture their appearance and voice” [19].

The importance and difficulties in maintaining memories of the deceased parent go hand in hand. While children desire voice recordings as a means of preserving memories, the creation of self-selected biographical sequences in the form of audiobooks allows dying parents to transcend time and shape their own culture of remembrance [18, 19]. Surviving children thus receive an authentic digital legacy whose interpretation, credibility, and reliability are strengthened by the parental voice recording. Research findings provide strong evidence of the potential benefits of digital legacy for children and for motivating and supporting a terminally ill parent to engage in this practice of digital legacy [17, 19–21]. A further study on the impact of Dignity Therapy on family members identified a positive experience with the legacy document. The digital legacy is regarded as a source of comfort and may potentially help to facilitate the bereavement phase [22]. However, one study highlights that research on legacy and areas of memory remains a complex and under-researched area within the social sciences [23]. To date, there is little research in this area. Considering these studies’ findings, it can be assumed that Reminiscence and the creation of a personal legacy represent an appropriate intervention for parents who are terminally ill and have dependent children under the age of 18 [17, 24].

As an intervention for terminally ill parents with dependent children, the recording of an audiobook as a legacy has been offered since the start of the project “Family Audiobook” in 2017. The Family Audiobook Association is a non-profit organization supported by fundraising and is free of charge. The family audiobook comprises several chapters organized chronologically, with a heading to each chapter given by terminally ill parents and their music or songs, such as sung lullabies. They also introduce the family audiobook to their family, talk about their recording experience, and explain the chosen title. The family audiobook comes with a small booklet with a table of contents and some photos.

This exploratory study examines how terminally ill parents and close persons perceive the audiobook and how satisfied they are with it.

## Methods

To investigate the potential of family audiobooks as a means of audio-graphic reminiscence of terminally ill parents for their dependent children under 18, an anonymised online survey was conducted.

### Study population and recruitment process

Study admission criteria for the recording of an audiobook were (1) patients diagnosed with life-limiting disease, (2) fluent in the German language, (3) 18 years of age or older, and (4) having at least one dependent child under the age of 18 years. The exclusion criterion was psychiatric impairment such as dementia, psychosis, severe depression, or diagnosed personality disorder.

Family Audiobook Association is a non-profit, voluntary organization, free of charge to participants and scientifically monitored. While the initial recruitment process came from the project team, the project group is now receiving an increasing number of interview requests from those meeting the criteria. This is due to a high level of media attention given to the final product, a family audiobook recorded by a terminally ill parent for their dependent children under the age of 18. The journey of the “Family Audiobook” project started in the pilot phase from 2017 to 2020, primarily in North Rhine-Westphalia. Since 2021, the project group has been accepting requests from all over Germany and neighboring countries. At the time of the online survey, over 350 terminally ill parents had recorded an audiobook. Almost all terminally ill parents (94%) have cancer; 78% of family audiobook recordings are made by women [25].

All family audiobook participants agreed to scientific monitoring and provided contact details to the Family Audiobook Association for this purpose. The Family Audiobook Association maintains a database of 234 email addresses. Invitations were only sent to those who provided an email address. The association sent out invitations to take part in an anonymous online survey and followed up with three reminders. This procedure ensured data protection agreements with families and guaranteed anonymity for participants. The research ethics committee of the University Hospital Bonn reviewed and approved the study (no.389/16).

### Data collection methods and research design

A snowball sampling procedure was used, whereby email recipients were requested to forward the link of the anonymous online survey to those who also had access to the family audiobook. The primary data was collected by using a standardized, mainly close-ended online survey accessible from 17.09.2023 to 30.11.2023. The quantitative online questionnaire consisted of 40 closed questions and 4 open-text input fields. The questionnaire was available in German.

To ensure an appropriate questionnaire length, several socio-demographic questions were excluded, thus allowing for the inclusion of more questions related to the research objectives. Some questions were only posed to patients who were terminally ill, as the questions pertained to the process and satisfaction with creating a family audiobook, specifically concerning the feelings of having done so or of having achieved it. Most of the questions focused on the initiation of the family audiobook, the listening experience, the content, and the sharing procedure. Additionally, participants were given the opportunity to provide information regarding the frequency with which children listened to the family audiobook and the perceived influence of the audiobook on the children. Further open-ended questions and text entry boxes allowed respondents to provide more detailed responses in their own words.

### Analysis

This is the first attempt to gain insight into the usage of family audiobooks as a means of reminiscing from the perspective of terminally ill parents and the ones left behind. In the absence of sufficient information about the contact persons for this study, the term “close persons” refers to a broad category of individuals, such as a diverse range of family systems and personal relationships. Due to small numbers, the category of “close persons” includes a diverse range of respondents, including partners, ex-partners, parents of patients, siblings, cousins, friends, bereaved children, and other relatives not further specified. Consequently, the anonymous quantitative data are divided into two groups of respondents: terminally ill parents and close persons.

The open-ended response fields were recoded and grouped. Content-related illustrative quotations were selected and grammatically corrected when necessary. The exploratory analyses entail frequencies and bivariate distributions in the context of cross-tabulations with the two groups of respondents. The analyses were performed using a statistical software platform (IBM® SPSS® 29.0 software platform).

## Results

From 17.09.2023 to 30.11.2023, the Family Audiobook Association sent an email to 234 addresses that included the link to the anonymous online questionnaire. Of the 234 emails sent, 214 email addresses were successfully reached by not receiving a reply of “email address not valid”. Nevertheless, the precise number of individuals reached remains uncertain, as the email cover letter requested email recipients to forward the online survey link at their initiative to those to whom the family audiobook is addressed. The aim was to reach as many as possible rather than to be representative. Finally, 186 respondents filled in the online survey.

**Fig. 1:**
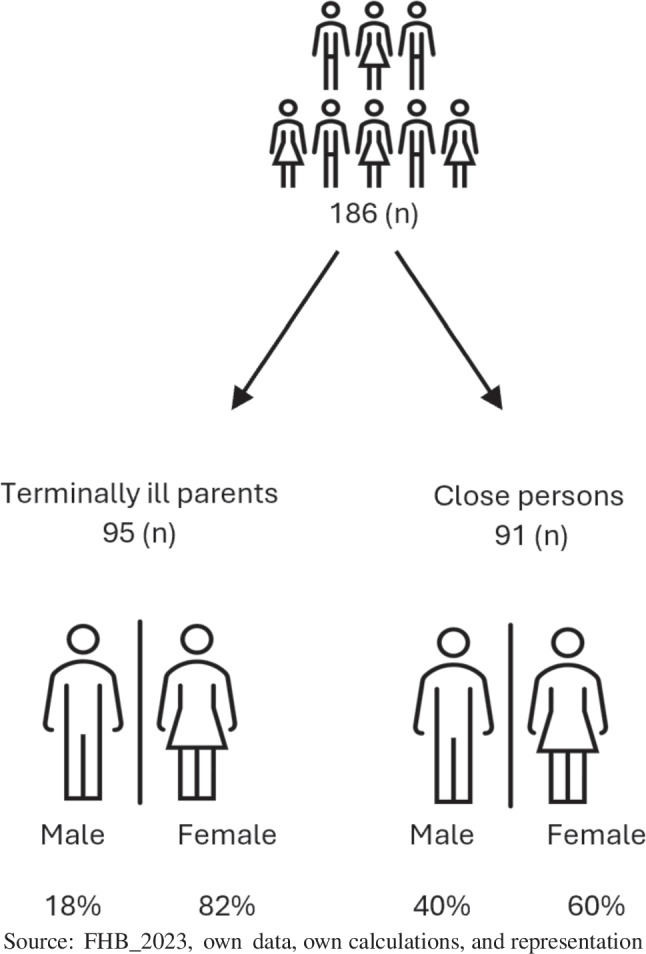
Sample size and distribution

Half of these 186 respondents are terminally ill parents with dependent children under the age of 18 (95), and the other half are close persons (91). Most of the terminally ill parents (82%) and 60% of the close persons are female (Fig. 1). The question regarding familial status was asked only to terminally ill parents themselves. The majority of the terminally ill parents are married (82%), 12% are in a partnership, and a minority are widowed, single, or separated.

### Terminally ill parents’ reflections on family audiobook

From the perspective of the terminally ill parent, it was of interest to ascertain whether the production and the possibility of passing on a family audiobook to their children is perceived as a burden. Table 1 reveals that feeling at ease was reported by a significant majority of respondents: 81% for having achieved it, 71% for having received it, and 76% for being able to pass it on. At the same time, a small proportion of terminally ill parents indicated on a five-point Likert scale that they would feel burdened by having reached it (5%), having recorded it (3%), or being able to pass it on to their children (3%) (Table 1).

**Table 1:** Terminally ill parents’ feelings about the family audiobook; percentages (*n* = 90)

	Having achieved it	Having received it	Being able to pass on
1 Burdens me	5%	3%	3%
2	0%	6%	2%
3	3%	7%	6%
4	11%	13%	13%
5 Eases me	81%	71%	76%

Source: FHB_2023, terminally ill parents’ responses only, own data, own calculations, and representation

### Passing on a family audiobook

When all respondents were asked who would give the reminiscence “family audiobook” to the children, 32% named the terminally ill parent, followed by the children’s partner or father at 54% and family members at 9%. “Other” and “don’t know” each accounted for 5% of responses. At the time of the survey, 38% of respondents stated that they did not know when they would pass on to the children. According to 24% of the respondents, the children have already received the family audiobook, a further 24% will receive it after the death of the terminally ill parent, and 12% will pass it on when the children reach a certain age.

### Use of family audiobook

It is important to note that the online survey was completed by many adults rather than children, thereby representing the perspectives of terminally ill parents and close persons who answered on their behalf. In light of this, based on 100% of all responses and in descending order, the most frequently mentioned person is a partner, followed by a family member or friend. Table 2 gives an insight into the study focus, whether and how often the audiobook is used. This question was posed to terminally ill parents and close persons (Table 2) and at the level of the children as an external assessment by the respondents (Table 3). Up to 60% of all respondents listened to the audiobook immediately after receiving it, more than 30% at least within six months, and 9% after 6 months. More terminally ill parents (68%) stated that they listened to the family audiobook immediately after receiving it. This was the case for 49% of responses from close persons. A multiple-response option was provided to identify potential accompanying persons while listening. This analysis is based on a scale of 100%, which represents the totality of 192 responses. The results indicate that 60% of respondents listened to the content alone, 16% with a partner, 13% with children, and 5% with parents.

**Table 2:** Post-received listening period and listening frequency; percentages

*Post-received listening period*			
	Terminally ill parents (*n* = 72)	Close persons (*n* = 75)	Total(*n* = 147)
Immediately upon received	68%	49%	59%
1 month after received	19%	11%	15%
1 to 6 months after received	11%	23%	17%
6 months to 1 year after received	1%	11%	6%
More than 1 year after received	0%	7%	3%
*Listening frequency*			
	Terminally ill parents (*n* = 72)	Close persons (*n* = 74)	Total(*n* = 146)
Often	1%	8%	5%
Sometimes	20%	45%	33%
Rarely	54%	38%	46%
Never	25%	8%	16%
*Feelings during listening*			
	Terminally ill parents (*n* = 72)	Close persons (*n* = 75)	Total (*n* = 147)
Very happy	15%	8%	12%
Happy	18%	9%	14%
Partly-partly	57%	57%	57%
Sad	7%	9%	8%
Very sad	3%	16%	10%

Source: FHB_2023, own data, own calculations, and representation

**Table 3:** Estimation of children’s listening frequency; percentages

	Terminally ill parents (*n* = 14)	Close persons (*n* = 28)	Total (*n* = 42)
Often	14%	11%	12%
Sometimes	29%	29%	28%
Rarely	36%	54%	48%
Never			0%
Don’t know	21%	7%	12%

Source: FHB_2023, own data, own calculations, and representation

The frequency of listening was measured by using a four-point scale, with responses ranging from “often” to “never.” Table 2 demonstrates that a total of 45% of the close persons and 20% of the terminally ill parents listen to the family audiobook “sometimes.” A total of 25% of terminally ill parents and 8% of close persons reported that they had never listened to the family audiobook at the time of the survey.

This raises the question, “How do you feel when you listen to the family audiobook?” Of all terminally ill parents and close persons, 57% indicated a state of emotional ambivalence, feeling partly sad and partly happy at the same time. The remaining 43% reported experiencing either (very) sad or either (very) happy to varying degrees (18% vs. 26%) while listening to the family audiobook. Terminally ill parents with dependent children under the age of 18 were more likely to report high levels of happiness (33%) than close persons (17%) (Table 2). In terms of the order in which respondents listened to the family audiobook, 69% selected the option of listening to it in a chapter-wise manner, while 33% listened to it from the first to the last chapter.

In those cases where the family audiobook has already been given to the children, 85% of the terminally ill parents and close persons confirmed that the children have already listened to it. On a four-point scale from “often” to “never,” 48% of terminally ill parents and close persons indicated that the children rarely listen to the family audiobook, 28% sometimes, and 12% often (Table 3).

#### External impression of influence on children

In terms of the impression of influence on the children, a spectrum of emotional reactions, from sadness to happiness, was reported by terminally ill parents and close persons, which went hand in hand with the different uses and influences on the grieving children.

“Two of the three children occasionally listen to the audiobook that my husband has recorded. However, this depends very much on their mood and stage of grief. The eldest doesn't want to listen to anything at all.” (close person)

“Mixed, one chapter the child didn't want to continue listening to. Now she listens to individual chapters more often, as I have made a part of it available on a Tonie [an audio player brand, authors’ note]” (close person)

“My son listens very attentively. And when friends come to play, it is also listened to.” (close person)

Some terminally ill parents who are facing the end of their lives listened to the family audiobook either during the production process or immediately after the finished audiobook was delivered. In cases where the family audiobook was finalised and made available to the children, a terminally ill parent wrote the following:

“I'm still here. That's why the kids don't hear it that often. But they know that when I'm no longer there, they can hear my voice. I think that's very important for the children. I'm sure it makes them sad too, but they're glad that I've recorded this audiobook. That way I'm always there somehow.” (terminally ill parent)

The respondents’ comments demonstrated that the process of remembering is strongly influenced by the experience of hearing the voice of the deceased parent while listening to the life events or lullabies.

“Very good, I listen to it again and again. Mum's voice is good.” (close person)

“My children are 5 and 7 years old. After their father died, they said at some point that they couldn't remember Dad's voice, so we listened to the audiobook. The 5-year-old was two years old at the time of my husband's death, he can't do much with it yet, but the 7-year-old asks about it from time to time.” (close person)

The respondents perceived the family audiobook as a valuable and cherished digital legacy, as well as an enriching form of remembrance. This is emphasized by the personal, carefully selected stories on the audiobook and the experience of hearing the voice of the dead or dying parent through the medium of the audiobook, which will, therefore, not be forgotten.

“On the positive side, I have the impression that it is seen as a ‘treasure’ for ‘later’.” (terminally ill parent)

#### Reasons for not listening to the family audiobook

23 of the 186 responses of terminally ill parents and close persons who had not listened to the family audiobook were asked to provide an explanation using a multiple-response scale and an option to write in their own words. The most frequently selected option (*n* = 13) was “other (please specify)”, followed by the option “need for distance”, which was the second most frequently selected option (*n* = 6). The aforementioned emotional aspect (6) is one of the reasons followed by “recordings have been stopped” or “have not yet been received” (*n* = 3). At the time of the survey, terminally ill parents and close persons provided several reasons for their decision not to listen to the recordings. These reasons also included their involvement in the recordings, the inability to contact the father, and the existence of a rule preventing the listening of the recordings until one year after the death influenced their decision not to listen. One terminally ill parent wrote: “Then you think of too many things you would have liked to have said or done differently.”

## Discussion

A review of the existing literature revealed a research gap in the field of digital audiobooks and their use by bereaved children [6, 19, 20], which might be due to a great deal of voluntary work [26]. Six years following the initiation of the project “Family Audiobook,” the first formal assessment was carried out to ascertain the use and the influence of the family audiobook created by terminally ill parents with dependent children under the age of 18. Therefore, it was of utmost importance to conduct a carefully designed and sensitive survey to gain novel insights into the use of family audiobooks. It is important to note that the results of this study are related to the fact that terminally ill parents proactively approached the Family Audiobook Association to have a family audiobook recorded.

The potential participants behind the email contact details were not known, nor were the final respondents. However, the anonymous online survey was completed by 186 respondents, 95 of whom were terminally ill parents and 91 close persons. The great majority of terminally ill parents indicated that they felt at ease with recording a family audiobook and then being able to pass it on to their children. Although the primary intended audience of the family audiobook is children, the survey results demonstrated that the terminally ill parent and close persons also listened to the family audiobook and provided answers for themselves. Many respondents expressed ambivalence when asked about their feelings when listening to the family audiobook. However, a greater proportion of parents with terminal illnesses reported feeling happy while listening than close persons. The responses of terminally ill parents and close persons to the survey question regarding the influence of the family audiobook on children exemplified the full spectrum of emotional reactions and their coping mechanisms observed in children, particularly between siblings, when engaging with the family audiobook. The respondents indicated that some children appear to experience less distress when listening than others. Some children avoid certain chapters, while others avoid the entire audiobook.

Leaving a narrative or memory for children is seen as important by both the bereaved and the terminally ill parent [8, 23]. The issues of digital legacy, rights, and ethics are also raised and discussed by the Digital Legacy Association (digitallegacyassociation.org, accessed 26/07/2024) [27, 28]. There are web tutorials for various formats to help with thoughtful implementation and to foster autonomy (digitallegacyassociation.org/for-the-public/, accessed 26/07/2024). The greatness and power lie in being the creator of memory content in one’s own voice. Additionally, professional support makes it possible to give the terminally ill parent a guide and horizon for the narrative flow [18, 29, 30]. Concurrently, it offers a helpful method of providing support to bereaved parents as a primary source of reminiscence. The findings of the study support those of previous literature. Many terminally ill parents who responded to the survey indicated that the provision of a family audiobook was perceived as a positive experience characterized by a certain sense of relief. Further study findings are also consistent with other research showing that a professionally supported strategy to reduce the burden by removing the responsibility of remembrance from the terminally ill parent in advanced stages and/or from the bereaved parent would be an important step forward [14, 15, 18, 26].

Finding the right time to pass on a legacy to children is another struggle, according to the literature [8, 19, 31]. In the project “Family Audiobook,” some parents who were approaching the end of their lives engaged their children in the production of an audiobook for the family. In doing so, they may have mitigated the burden on others in the care system. Moreover, proactively communicating and documenting the date of the audiobook handover to the children may reduce the burden on the bereaved parent, as these decisions are clarified and guaranteed. This is particularly relevant when one considers that studies have identified this aspect as a substantial source of stress for bereaved parents [18, 19].

The respondents to this study highlighted the significance of maintaining the voice in family audiobooks, emphasizing its role in keeping reminiscences alive. This finding aligns with the conclusions of other studies in this field [8, 15, 18, 19]. The digital legacy in the form of an audiobook allows especially dependent children under the age of 18, to engage in memory work at their own pace and independently of the grieving parent. The external statements regarding the influence of the family audiobook on children indicate that a family audiobook has the potential to be a relevant resource in situations where children are experiencing grief and use the family audiobook as a coping strategy throughout the grieving process. The results of this study also highlight a great ambivalence in the turmoil of the respondents’ emotional worlds. The audiobook keeps memories alive; even so, it would be better to “make memories yourself”.

This research indicates that there are as well opportunities as risks associated with leaving a legacy of the dying parent in the form of a family audiobook [28]. It is essential to identify any criteria that would rule out the use of a biographical intervention. It is noteworthy that there may be characteristics that differentiate between individuals who respond to such interventions and those who do not, as evidenced by the findings of the study on Life Review [32]. The Life Review study identified three key factors contributing to a lack of response: fear of the future, family conflicts, and practical problems [32]. It can be assumed that terminally ill parents who hold a positive outlook on their lives, experience a sense of everyday satisfaction, and maintain a balanced lifestyle may be more likely to benefit from the intervention. It may also be assumed that these individuals are less likely to experience feelings of loneliness, anxiety, and depression. Conversely, terminally ill parents with a history of traumatic or negative experiences may initially be hesitant to participate in such an intervention. It is, therefore, necessary to consider which individuals might be expected to benefit from this intervention. Following the heuristic model proposed by another study, reminiscence may also result in adverse outcomes, including heightened rumination, anxiety, and unresolved conflicts [30]. In addition to the family perspective, this approach may assist healthcare professionals in mitigating the stress associated with providing end-of-life care for terminally ill parents with dependent children under the age of 18 [6, 26].

In the future, new technologies will bring other forms of digital afterlife into question. In addition to chatbots, these now include virtual realities in which the bereaved children can immerse themselves and interact with their deceased parent, even holding entire conversations [33, 34]. In addition to the future, new technologies and the emergence of other forms of digital afterlife will give rise to new questions, some of which will build upon existing ones. One such question remains for further examination in the context of digital reminiscence. In all cases, research utilizing a child-appropriate questionnaire would enhance the understanding not only of the long-term usage but also the challenges, overall effects, and emotional risks associated with digital reminiscences for children.

## Strengths/limitations

The strength of this study lies in the visualisation of satisfaction with the realisation and receiving of the family audiobook on the part of terminally ill parents, which was achieved through an inquiry. Concurrently and in nearly identical proportions, the close persons, mainly family members, responded to the online questionnaire. The results of this study indicate that family audiobook usage varies considerably, with a tendency toward infrequent listening. This is likely due to the nature of confronting the death of a parent. Consequently, the findings of this study found that the transfer of ownership and responsibility for a family audiobook is a highly emotional and sensitive process. The ambivalence becomes visible on the part of the family members and can now be better addressed and supported. However, this requires sensitive further research into the influences on the children left behind. Especially the influences on the children are expressed by third parties, this study is limited. This approach lacks a longitudinal study, particularly on the impact of family audiobooks from the children’s perspective.

The absence of a list of respondents for full surveys or representative sampling prevents the study from being generalisable. A further limitation is that the anonymised responses cannot be grouped into families. This implies that only a single individual may have responded to one audiobook, while in other cases, multiple individuals may have responded to one audiobook. Additionally, some individuals have been contacted but did not answer the survey since they have not yet received the audiobook. The diverse motivations for participation in the survey also introduce bias and a limitation to the studies. The exploratory nature of this study has an impact on the depth of the data, as there is limited previous research in this area. Further accessibility could be achieved by using multilingual questionnaires to encourage the participation of people with non-German language skills. This also applies to the measurement instrument, which mainly reaches digitally connected people. As previously noted, a significant number of terminally ill parents involve their children or partners during the recording process. Furthermore, (semi-structured) in-depth interviews could provide additional insight into the influence of the creation process on the owner of a family audiobook. A mixed methods approach would address some of these limitations but is more resource-intensive.

## Supplementary Information

Below is the link to the electronic supplementary material.

Supplementary file1 (DOCX 32 KB)

## Data Availability

Data and material are not openly available due to reasons of sensitivity and are available from the corresponding author upon reasonable request.
